# Examination of PDK1/AKT/mTOR transcription and exosomal mRNA levels in human glioblastoma cell line treated with a combination of temozolomide and hesperidin

**DOI:** 10.1007/s00210-025-04137-4

**Published:** 2025-05-15

**Authors:** Sidika Genc, Betul Cicek

**Affiliations:** 1 Faculty of Medicine, Department of Medical Pharmacology, Bilecik Şeyh Edebali University, Bilecik, 11230 Turkey; 2 Faculty of Medicine, Department of Physiology, Erzincan Binali Yildirim University, Erzincan, 24100 Turkey

**Keywords:** AKT, mTOR, miR- 9, Glioblastoma, T98G

## Abstract

The most malignant type of tumor in the brain is high-grade gliomas. Glioblastoma (GB), a grade 4 glioma, has the lowest 5-year survival rate and is associated with poor prognosis. An important signaling pathway involved in the pathogenesis of GB is the mammalian target of rapamycin (mTOR). Therefore, our study aimed to investigate how exosomes obtained from GB cells applied with different doses of hesperidin (HSP) affect miR- 9 and change the PDK1/AKT/mTOR pathway. For this purpose, T98G cells were treated with different doses (5, 10, 25, and 50 µg/mL) of HSP in combination with temozolomide (TMZ- 10 µg/mL). At the end of 24 h, cell viability, flow cytometry, and biochemical tests were performed. Additionally, exosomes were isolated from cells belonging to the control, TMZ, and high-concentration TMZ-HSP groups. miR- 9, PDK1, PTEN, AKT- 1, Bax, Bcl- 2, and Caspase 3 genes were expressed in both application groups and exosomes belonging to these groups. HSP was found to reduce the viability of GB cells significantly. The viability was significantly reduced, especially in the TMZ-HSP 50 µg/mL group. Depending on the dose, there was a significant increase in the LDH level and oxidative stress level. The apoptosis level was approximately 26% in the TMZ-HSP 50 µg/mL group. Along with all this, gene expressions changed at the exosomal level, and miR- 9 and miR- 146 levels increased. Similarly, it changed the expression of proteins related to the PDK1/AKT/mTOR signaling pathway at the exosomal level (*p* < 0.05). In conclusion, the TMZ-HSP combination showed anticancer effects in T98G cells, influenced exosome profiles, and appeared non-toxic and potentially beneficial to healthy cells, highlighting its potential therapeutic value.

**Graphical Abstract:**

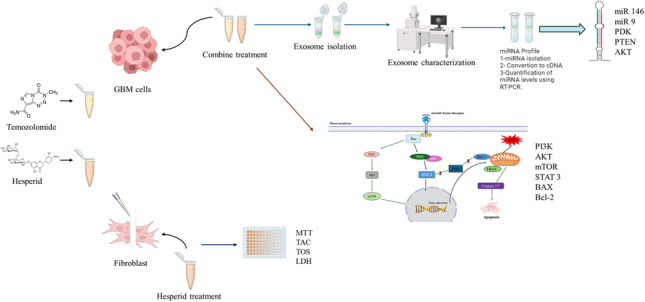

## Introduction

The most prevalent and most malignant primary brain tumor is known as glioblastoma (GB), with an annual incidence of fewer than 10 per 100,000 persons (3.2 in the USA, for example (Tamimi & Juweid 2017). The prognosis and mortality rate for GB remain poor despite intensive research and efforts to create appropriate and effective treatments. The conventional treatment for just-diagnosed GB patients involves surgical resection, succeeded by radiotherapy (60 Gy in 30 segments) with simultaneous oral temozolomide (TMZ), followed by six cycles of adjuvant therapy In TMZ-resistant GB tumors, various molecular pathways are frequently dysregulated, including nuclear factor kappa light chain enhancer of activated B cells (NF-ƘB), p53, and JAK-STAT (Lee 2016). The RNA molecules known as miRNAs, or microRNAs, are small (18–25 nucleotide length) non-coding RNAs that attach to the target mRNA’s 3′UTR region. miRNAs have been particularly promising because they may regulate the expression of the targeted genes and the quantities of essential proteins (Brower et al. 2014). One of the primary sources of these RNA biomarkers is newly discovered and highly researched extracellular vehicles (EVs) (Cocks et al. 2022). Due to the physiologically active chemicals they contain, exosomes, which are the smallest EVs (diameter 50–150 nm), are consistently studied (Genc et al. 2022).

Exosomes are a valuable source of biomarkers in bodily fluids because they additionally transport many kinds of macromolecules, such as particular proteins and RNAs (Borlongan & Wang 2023; Genç et al. 2021). Their intracellular cargo could promote numerous processes indicative of malignancy, including enhanced cell viability, chemoresistance, angiogenesis, and activation of cancerous signaling pathways such as the Signal Transducers and Activators Of Transcription (STAT) and SRY(sex determining region Y)-associated HMG (high- mobility-group)-box (SOX2) pathways (Wu et al. 2023).

The essential function of mTOR signaling in cell hemostasis is particularly substantial in the brain. Protein translation control by mTORC1 has been demonstrated to regulate the plasticity of synapses, memories, and cognition (Laplante & Sabatini 2012; Neasta et al. 2010). PI3 K signaling has been modulated via mTOR, and current research indicates that it could represent a potential molecular target in glioblastoma patients (Jhanwar-Uniyal et al. 2015). The PDK/AKT/mTOR pathway is crucial for controlling cellular apoptosis, survival, metabolism, and differentiation. miR- 9, miR- 124, miR- 132, and miR- 134 work to suppress NF-ƘB and AKT/mTOR signaling pathways to limit tumor growth indirectly (Juan Lu et al. 2018; Qin et al. 2016; Xie et al. 2018; Zhang et al. 2018). Tyrosine kinases, G-protein-coupled receptors, or the oncogene RAS can all activate the PI3 K signaling pathway.

HSP belongs to the flavanones family of flavonoids and has been associated with broad-spectrum usefulness in preventing the development of fatal diseases such as cardiovascular disease, neurodegeneration, and carcinoma. HSP’s anticancer benefits have been linked to its antioxidant and anti-inflammatory properties. HSP interacts with various known cellular targets and inhibits cancer cell proliferation by triggering apoptosis and cell cycle arrest. Moreover, evidence points out that it has a promising effect in suppressing tumor cell metastasis and angiogenesis (Ahmadi & Shadboorestan 2016; Alshatwi et al. 2013; He et al. 2017; Li & Schluesener 2017). HSP is also suggested to prevent chemoresistance (Febriansah et al. 2014). Although there are many studies in the literature on HSP, there is no other study investigating how the TMZ-HSP combination is affected both at the exosomal level and at the cellular level.

In our current study, we applied HSP combined with temozolomide on the GB cell line. Our study aimed to examine both the anticancer activity of HSP and its effect on exosomal miRNA levels. For this purpose, we examined miR- 9–3, PDK, PTEN, AKT- 1, Bax, Bcl- 2, and Caspase 3 gene expression from both the cells of the control and treatment groups and the exosomes of these groups. In addition, we supported our study with cell viability, apoptosis, and biochemical analyses. This study will be presented in the literature as the first study to examine the gene expression of exosomes obtained from T98G on the mTOR pathway and to examine the change of HSP on the T98G system at the exosomal level.

## Materials and methods

### Glioblastoma and fibroblast cell lines

The GB cell lines T98-G and human Dermal Fibroblast (HDFn) were purchased from ATCC and provided by study participants at Bilecik Şeyh Edebali University (Bilecik, Turkey). In brief, the cells were suspended in a fresh medium (DMEM), 15% FBS, and 1% antibiotic suspension (penicillin, streptomycin, and amphotericin B). The cells were then cultivated in a CO_2_ incubator under suitable conditions (5% CO_2_; 37 °C) in 25 cm^2^ cell culture flasks (Corning, Corning, NY, USA) (Yeni et al. 2023).

### Exosome isolation and characterization

#### Exosome isolation

T98G cell lines were plated in a six-well plate to collect treatments of GB-derived exosomes. Two milliliters of complete medium was supplied after 70% confluency in a cell culture plate (Corning, USA). As previously reported, the Total Exosome Isolation Reagent (Invitro-genTM—Cat. 4,478,359, Waltham, MA, USA) procedure for efficient exosome isolation was employed. Briefly, conditioned medium and reagent (2:1) were combined and incubated overnight at 2 °C to 8 °C. Briefly, conditioned medium and reagent (2:1) were combined and incubated overnight at 2 to 8 °C. Following the incubation process, the samples were centrifuged at 10,000 g for 1 h at two to 8 °C to extract the exosomes from the bottom of the tube (Yeni et al. 2023).

#### Scanning electron microscope (SEM)

The outcomes of an SEM, or scanning electron microscope, were received from the Atatürk University DAYTAM center. For SEM imaging, 20 µL of exosome sample was put onto grids and lyophilized for 1 h using freeze-drying. Grids of molecules were Au-coated, and micrographs were obtained at 15 kVn using SEM equipment (Zeiss Sigma 300, Germany) (Yeni et al. 2023).

### MTT (3-(4,5-dimethylthiazol- 2-yl)− 2,5-diphenyltetrazolium bromide) assay

Immediately following 24 h of HSP treatment, each well plate received 10 µL of MTT solution (1:10). Following 4 h of incubation, 100 µL of DMSO (dimethyl sulfoxide) solution was added to each well. The optical density of the dissolved formed crystals of formazan has been measured at 570 nm using the Multiskan™ GO Microplate Spectrophotometer reader (Çiçek & Genç, 2023).

### Total oxidant status (TOS) and total antioxidant status (TAS)

TOS and TAS have been assessed by spectroscopic measurements (MultiskanTM GO Microplate Spectrophotometer reader, Waltham, MA, USA) according to instructions by the manufacturer (Rel Assay Diagnostics® Company, Gaziantep, Turkey). The well absorbance was measured at 590 nm for TOS and 660 nm for TAS (Yeni et al. 2023).

### Lactate dehydrogenase (LDH) assay

LDH is a superior metabolic measure of cell viability due to necrotic cells releasing it. Applying an LDH kit made it possible to assess how different treatments affected the LDH activity of T98G cells (Fig. 2A). The LDH assay was carried out with a commercially available test kit from Cayman Chemical Co., Ltd. (Ann Arbor, MI, USA). The cell culture medium was briefly centrifuged at 400 g for a duration of 5 min at the appropriate temperature. A volume of 100 L supernatant has been added to a 100 L reaction solution (LDH Assay Buffer, LDH Substrate Mix), then incubated at room temperature for 30 min with lightly shaking on an orbital shaking device. Ultimately, the absorption intensity was measured at the wavelength of 490 nm (Yeni et al. 2023).

### Human mTOR (mammalian target of rapamycin) ELISA

Standards or samples have been previously added to Micro ELISA plate wells with an antibody specific to human MTOR. After that, each microplate well is successively added to and incubated with the biotinylated detection antibody designed to be typical for Human MTOR. The free elements are removed by washing. The substrate solution is applied to each well. The only wells to display blue correspond to those containing Human MTOR, biotinylated detection antibody, and Avidin-HRP conjugate. The enzyme–substrate interaction is stopped when the stop solution is added, and the color changes to yellow. Optical density (OD) has been determined via spectrophotometry at 450 nm (Wang et al. 2013).

### Flow cytometer

HSP-treated T 98 cells were digested with trypsin (without EDTA). PBS was used to wash the cells, and then 1–5 × 105 cells were collected. The following steps are part of the Annexin V-FITC cell apoptosis detection kit (cat. no. KGA108; Nanjing KeyGen Biotech Co., Ltd., co.), which were carried out according to the manufacturer’s instructions. A total of 500 µL binding buffers were added to suspend the cells. Then, 5 µL Annexin V-FITC and 5 µL PI were mixed by adding, respectively. The examination was conducted in the dark at moderate temperatures for 10 min. A flow cytometer for observational detection (A00 - 1–1102; Beckman Coulter, Inc.) was utilized. The apoptotic percentage (the fraction of early and late apoptotic cells) was estimated (Rieger et al. 2011).

### Gene expression analysis

Utilizing Roche Mannheim’s High Pure RNA isolation kit, total RNA was isolated from the exosomes. Absolute methanol was used to dissolve the exosome capsule. Special filters were used to remove the mRNA. We used specific primers and a high-capacity first-strand cDNA synthesis kit for RT-PCR (AMW) made by Roche (Darmstadt, Germany) to conduct a real-time polymerase chain reaction (PCR). The primer sequences are described below (Roche; Darmstadt, Germany) (protocol: an initial step of 94 °C for 5 min followed by 40 cycles of 94 °C for 30 s, 56 °C for 45 s, and 72 °C for 60 s). The findings have been defined as variations in relative mRNA expression (fold changes) between the experimental and control groups. We normalized the mRNA expression of target genes to beta-actin (reference control gene) using the ^ΔΔ^Ct method (Genc et al. 2023).

### Analysis of microRNA expression

Cell exosomes were used to extract total miRNA. A high-capacity miRNA reverse transcription kit (TaqMan microRNA reverse transcription kit, Thermo Fisher Massachusetts, ABD) was used to create complementary DNA (cDNA) from total miRNA. TaqMan microRNA assays and particular mature miRNA primers, whose sequences are described below (Roche; Darmstadt, Germany), were used for real-time PCR microRNA analysis (protocol: initial step of 94 °C for 5 min followed by 40 cycles of 94 °C for 30 s, 56 °C for 35 s, and 72 °C for 30 s). By applying the ^ΔΔ^Ct method, as previously reported, we normalized the miRNA expression of the target miRNAs to the RNU6 reference control (Genc et al. 2023).

### Wound healing assay

HSP’s effect on healthy cells was studied using human dermal fibroblast cells. Cells were grown in the abovementioned process, and a wound line was created. The wound line thus created was treated using different dosages of HSP. Every 4 h, photographs were taken to identify when the initial wound line closed. After 48 h, it was established that the wound line in the HSP 50 µg/mL group was nearly invisible. The experiment was termed off after 48 h. The markers of cytotoxicity and oxidative stress were studied.

### Statistical analysis

The statistical analysis was conducted utilizing the SPSS 22.0 application and a one-way analysis of variance (ANOVA), along with Tukey’s HSD for post hoc comparisons. For each analysis, the statistical limitation was established at *p* < 0.05.

## Results

### Exosome characterization

#### SEM

**Fig. 1:**
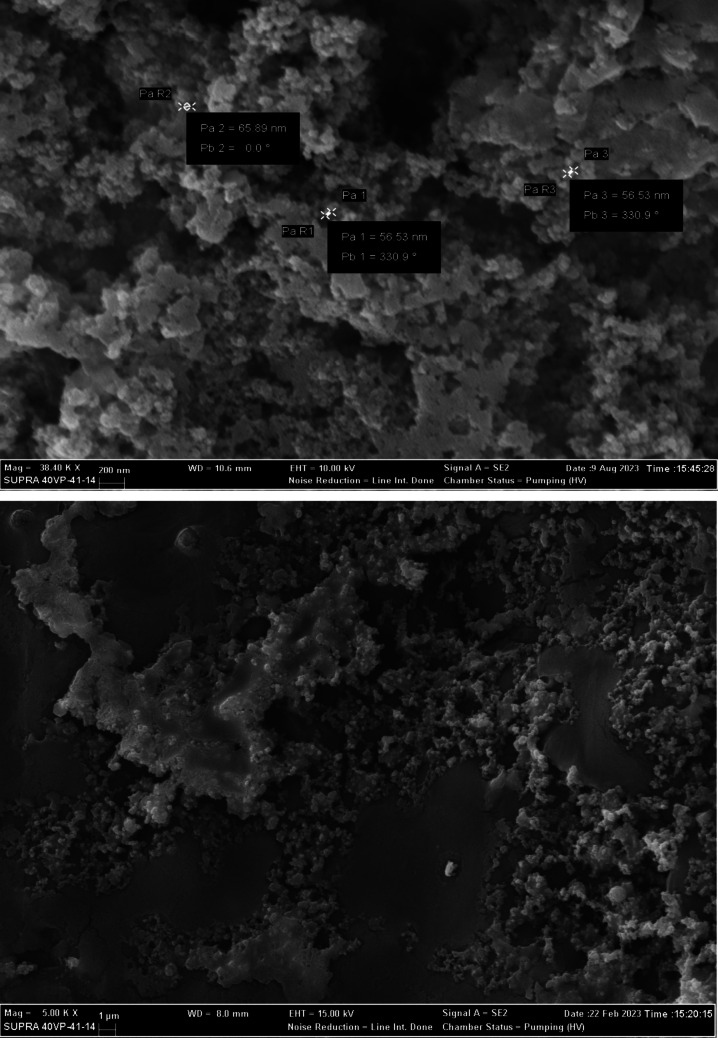
SEM examined exosomes. The white arrow shows particle size. Pa: particle size in nm, P: particle, Pa R: particle radius, and Pb: particle angle (The Carl Zeiss Evo 40 SEM (Jena, Germany) was used to capture the images)

To confirm the isolation of the exosomes we obtained, we examined the exosome size using SEM to validate our exosome isolation method. We observed that the number of exosomes obtained was quite dense, and we captured images of round-shaped microparticles with diameters in the reference range of 30 to 150 nm in the exosome-rich fraction (Fig. 1).

### Evaluation of T98G cell viability by MTT and LDH assay

**Fig. 2:**
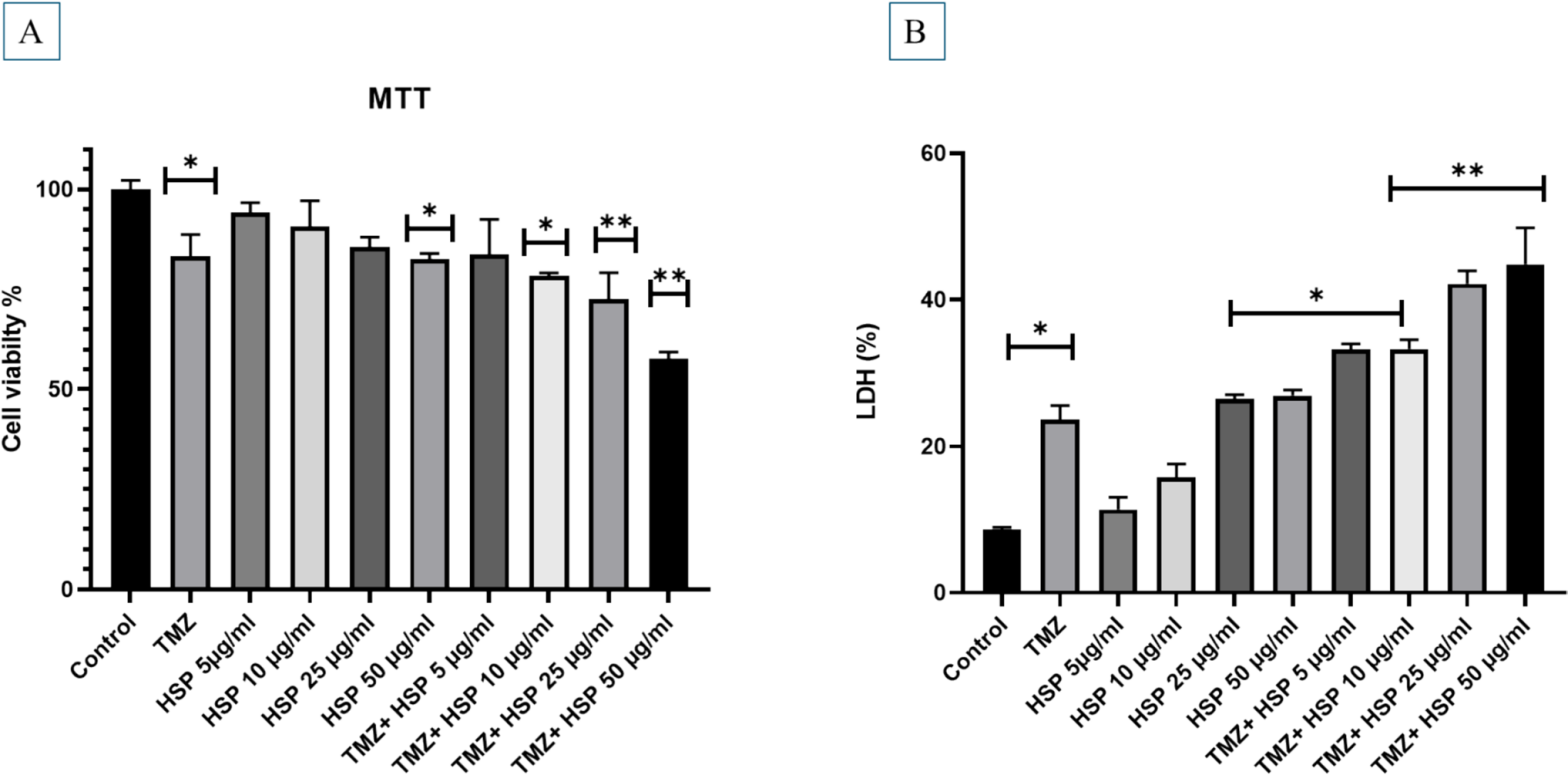
**A** The MTT assay was utilized to determine cell viability (*n* = 12)—the consequences of TMZ, HSP, and TMZ combined on the viability of T98G cells. **B** Impact of TMZ and TMZ + HSP on LDH Activity in T98G Cells (*n* = 6). LDH activity has been determined in cells that were treated with TMZ (10 g/mL), HSP (5,10,25, and 50 g/mL), and combinations of TMZ + HSP for 24 h in 96-well plates. The average of three different experiments is used to represent the results. Meaningful statistically: *p < 0.05; **p < 0.01

By employing the MTT assay 24 h after treatment, the impact of different treatments on T98G cell viability was evaluated (Fig. 2). Cell viability for the control (negative control) was expressed as a percentage of control for all other treatments and was assumed to be 100%. HSP did not substantially impair cell viability, even in low doses. Nevertheless, cells treated with TMZ 10 g/mL for 24 h saw a 17% decline in viability (*p* < 0.05). The combination of TMZ and HSP was found to have a more significant effect on cell viability. Therefore, treatment of cells with TMZ-HSP (25 µg/mL) for 24 h reduced their viability to 28% (*p* < 0.05). In contrast, treatment of T98G cells with TMZ-HSP (50 µg/mL) decreased their viability (42%) and caused a significant decrease (*p* < 0.01).

Figure 2A illustrates the estimated LDH production of the cells treated as a percentage of the reference (identified as 100%). Treating cells with only low doses of HSP did not affect their LDH activity. However, an increase in LDH activity, correlated with cell death, was demonstrated in cells treated with a combination of TMZ- (10 µg/mL) (*p* < 0.05) and cells treated with TMZ-HSP (10, 25, and 50 µg/mL) for 24 (*p* < 0.05 and *p* < 0.01), respectively. According to these findings, T98G and HSP significantly enhanced the active medication’s cytotoxic activity even at doses below the active drug range.

### Evaluation of T98G cell apoptosis effect of TMZ and HSP

**Fig. 3:**
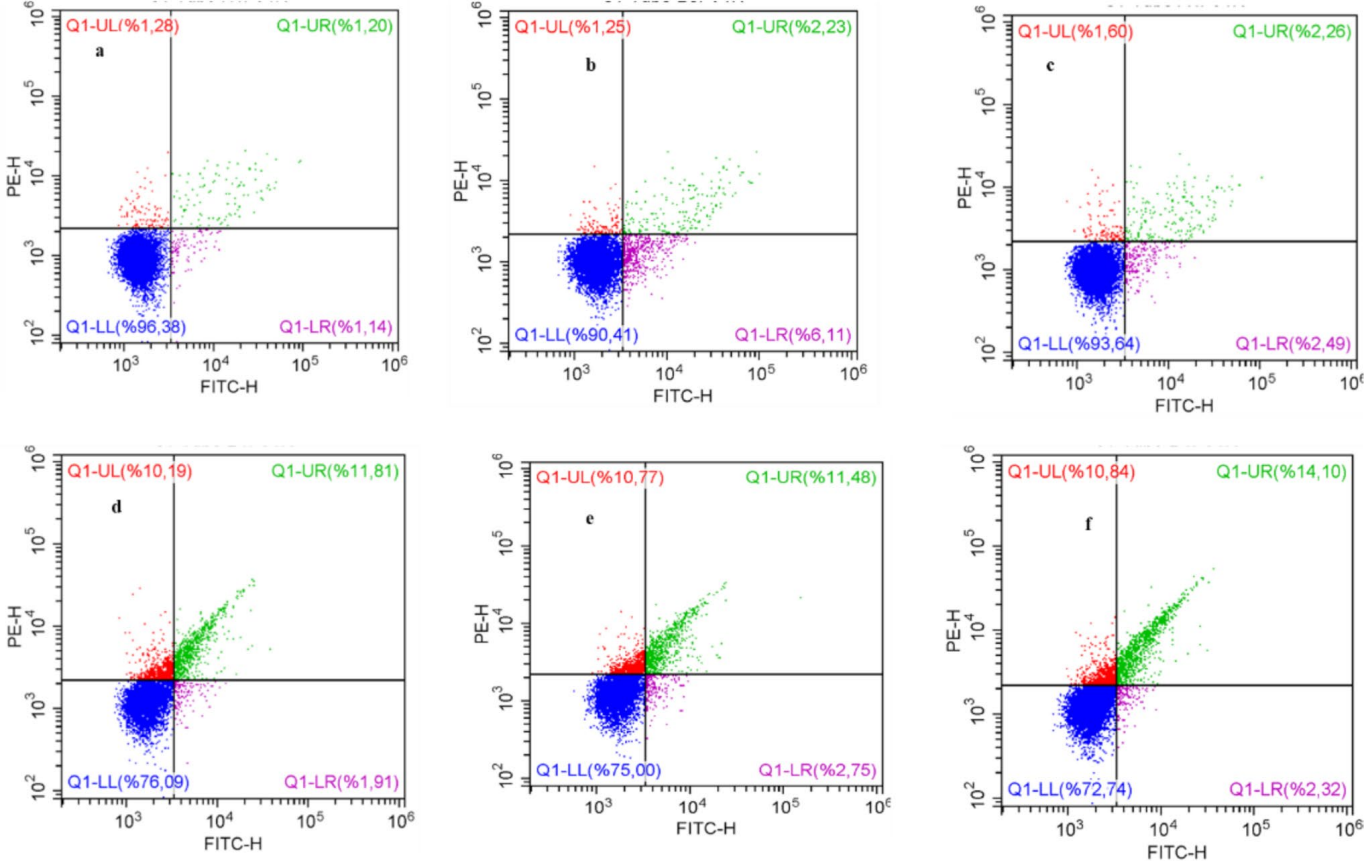
Live, early, late apoptotic, and necrotic cell populations were distributed in T98G cells after treatment with TMZ and HSP for 24 h. Apoptotic cells positive for Annexin V can be seen in the lower right quadrant, and dead cells positive for both annexin and PI can be seen in the upper right quadrant. Both stains show negative results in healthy cells (lower left quadrant). **a** Control, **b** TMZ 10 µg/mL, **c** HSP 50 µg/mL, **d** TMZ- HSP 10 µg/mL, **e** TMZ—HSP 25 µg/mL, and **f** TMZ- HSP 50 µg/mL. The symbols for statistical significance are *p < 0.05 and **p < 0.01

This study attempted to characterize the type of death that causes decreased viability on the T98G cell line. For this purpose, Annexin V/PI double staining was used to measure the percentages of live, early/late apoptotic, and necrotic cells. Flow cytometric graphs can be seen in Fig. 3. Low concentrations were ignored according to MTT results. In the examination performed at high TMZ and HSP concentrations (50 µg/mL), we found that apoptotic death occurred at low levels. It was shown to induce TMZ-HSP (10, 25, and 50 µg/mL) groups. Apoptotic cell percentages were found to be 23.91%, 25%, and 27.26%, respectively. It was discovered that cellular viability declined substantially higher in the combined groups, which is identical to our MTT findings.

### The effect of TMZ and HSP on T98G cells redox state

**Fig. 4:**
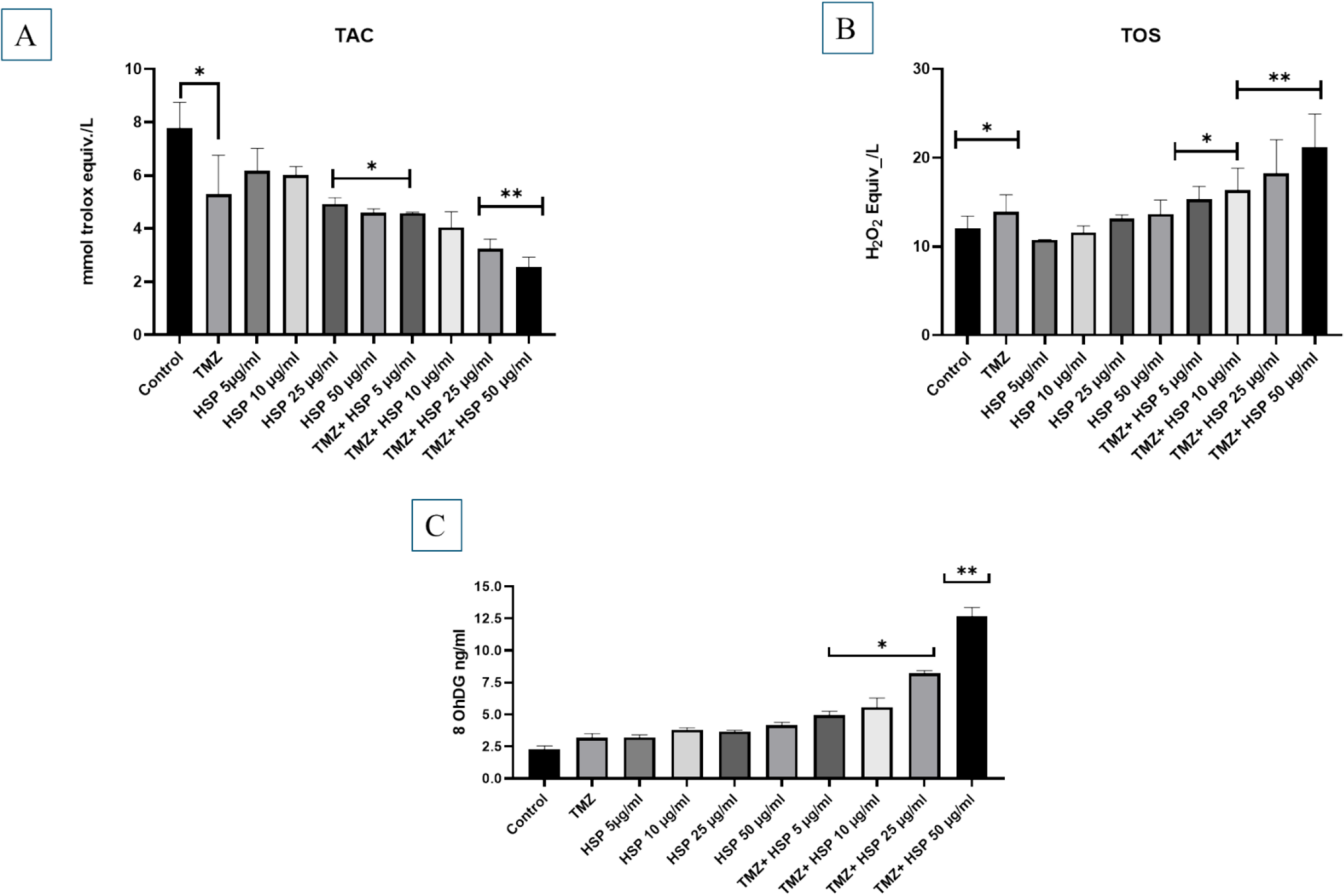
**A** Effect of the combination of TMZ and HSP on T98G cell TAC (*n* = 6). **B** Effect of the combination of TMZ and HSP on T98G cell TOS (*n* = 6). **C** Effect of the combination of TMZ and HSP on T98G cell 8-OhDG (*n* = 6). Meaningful statistically: *p < 0.05; **p < 0.01. The symbols for statistical significance are *p < 0.05 and **p < 0.01

The control group’s T98G cell TAC value, determined spectrophotometrically, was 7.78 mmol Trolox equiv/L (Fig. 4A). HSP treatment at low concentrations did not effect on these cells’ TAC. However, treatment with TMZ + HSP markedly reduced the antioxidant state of T98G cells in a concentration-dependent manner (Fig. 4).

In contrast with the TAC findings, the combination of TMZ + HSP therapy (Fig. 4B) was observed to elevate T98G cell TOS levels in a concentration-dependent manner. Treatment with TMZ + HSP 50 µg/mL exhibited the most noticeable effects (*p* < 0.01).

8OHdG is one of the most prevalent types of reactive oxygen species (ROS) degradation and is frequently employed as a biomarker for oxidative stress. The 8-OHdG biomarker was utilized in our work to demonstrate that the TMZ-HSP combination caused oxidative DNA damage in T98G cells. Cellular damage gradually increased in the treatment group, whereas the 8OHdG level was 2.32 pg-mL in the control group. Like the previous data, the TMZ-HSP 50 g/mL group showed the most notable result. The results were found to be particularly significant (*p* < 0.05 and *p* < 0.001) (Fig. 4C).

### Effect of T98G cells treated with TMZ-HSP on mTOR signaling pathway

The primary control of cell growth and division under appropriate circumstances is mTOR. On the other hand, tumor cells with improperly activated mTOR convey signals, causing tumor cells to proliferate and spread to healthy tissues. When the mTOR results of the control group in our study were examined, they were found to be relatively high. As the doses of the treatment groups increased, a decrease in the mTOR signal occurred. The most effective result occurred in the TMZ-HSP 50 µg/mL group *p* < 0.05 and *p* < 0.001) (Fig. 4D).

### RT-PCR results of T98G cells treated with TMZ-HSP

**Fig. 5:**
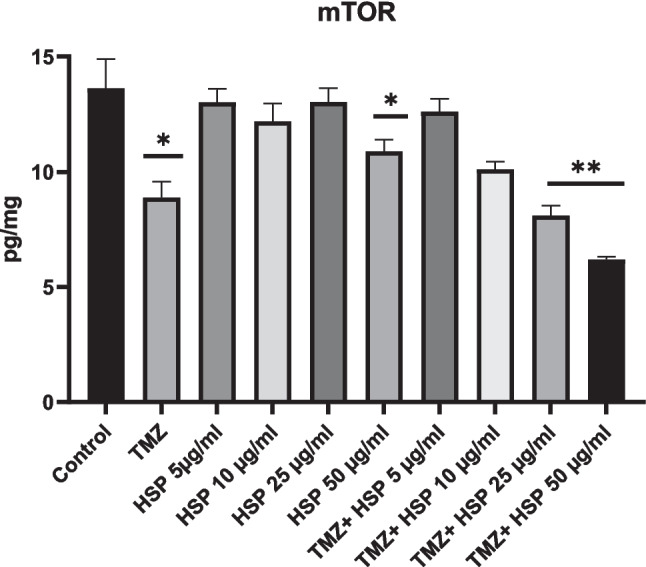
Effect of the combination of TMZ and HSP on T98G cell mTOR (*n* = 6). Meaningful statistically: *p < 0.05; **p < 0.01. The symbols for statistical significance are *p < 0.05 and **p < 0.01

**Fig. 6:**
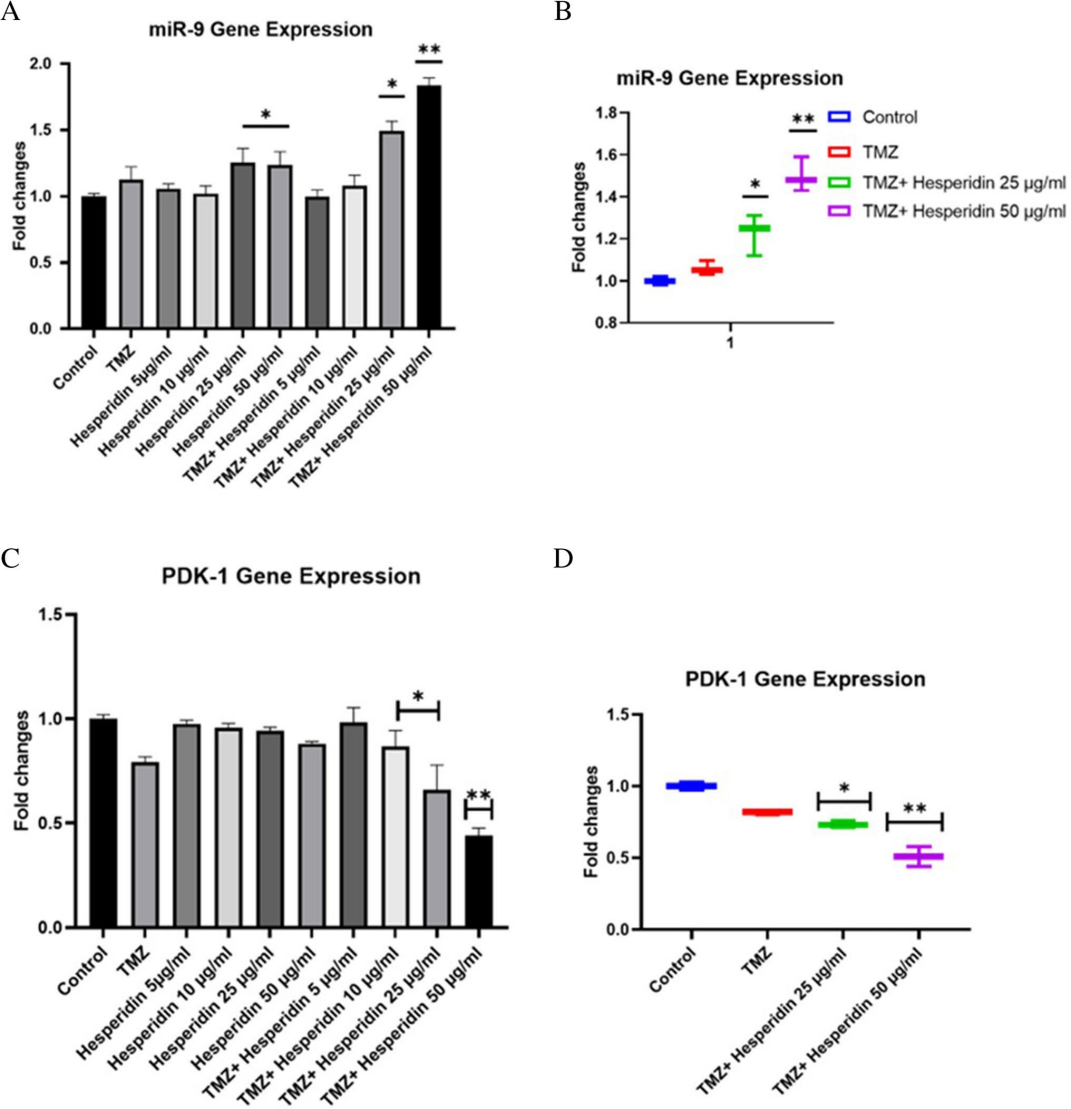

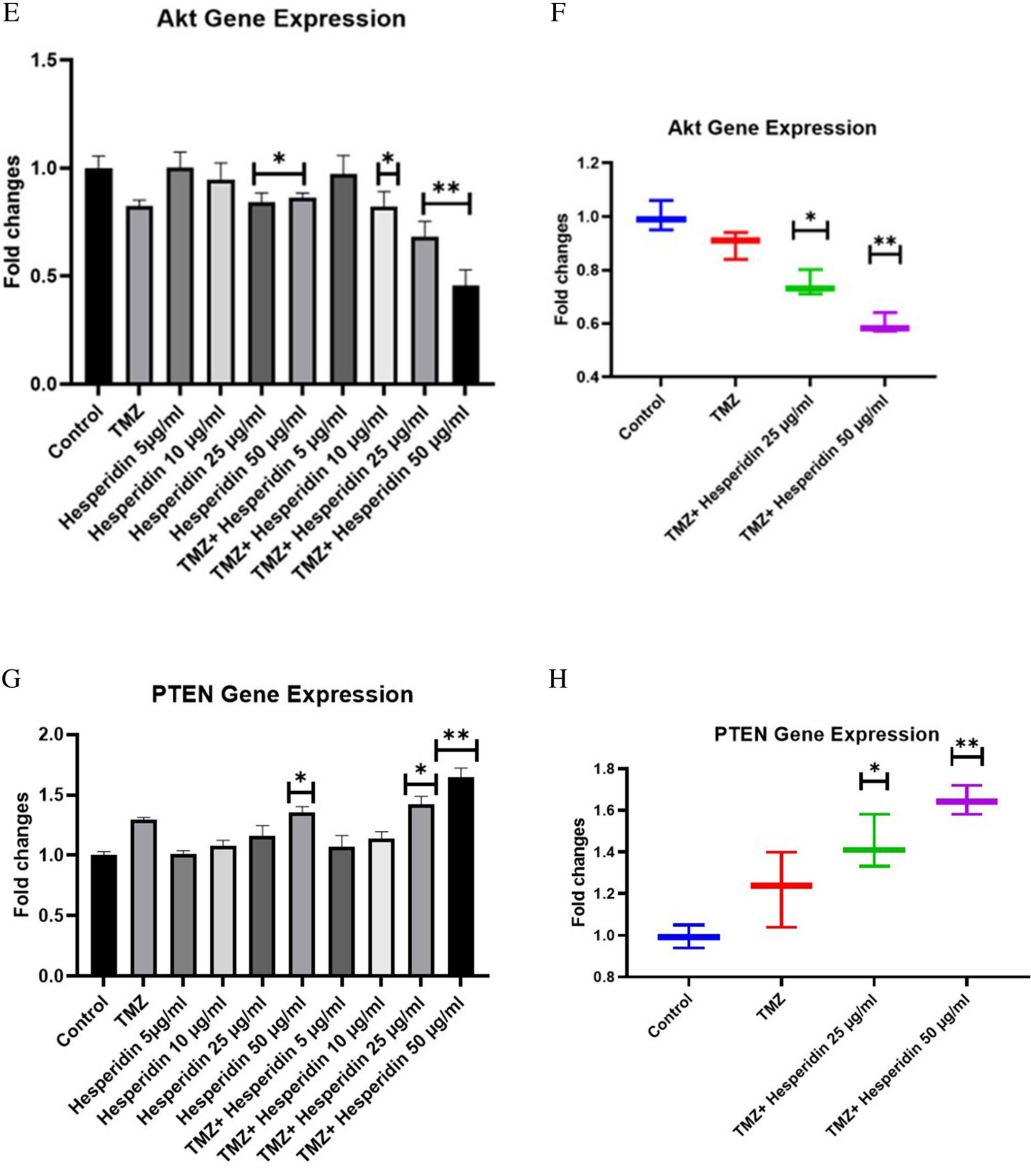
**A** miRNA9 gene level of treatment groups. **B** miRNA- 9 gene level of exosomes obtained from treatment groups. **C** PDK- 1 gene level of treatment groups. **D** miRNA9 gene level of exosomes obtained from treatment groups. **E** AKT gene level of treatment groups. **F** AKT gene level of exosomes obtained from treatment groups. **G** PTEN gene level of treatment groups. **H** PTEN gene level of exosomes obtained from treatment groups. Experiments were performed in triplicate, and the results are given as average. Meaningful statistically: *p < 0.05; **p < 0.01

RT-qPCR results showed that TMZ-HSP could improve the expression level of miR- 9 (Fig. 5A). The expression of miR- 9 following treatment with TMZ-HSP was significantly different in T98G cells. Following 50 μg/mL TMZ-HSP treatment, miR- 9 expression was upregulated. This indicated that TMZ-HSP could promote the expression of miR- 9. Additionally, a change in the mir- 9 level was observed at the exosomal level (Fig. 5B). The miR- 9 level in the exosomes of the control group was accepted as 1, just like the control group. Although the gene level was low compared to normal cells, the miR- 9 level was upregulated compared to exosomes from the control group. These results will be the first evidence to show that the miR- 9 exosome level may change with treatment with HSP. In contrast to miR- 9, the level of miR- 146 - 5p gene expression increased. Although the increase is minor in comparison to our other analyses, it confirms our treatment group’s findings (Fig. 6) (*p* < 0.05; **p < 0.01).

**Fig. 7:**
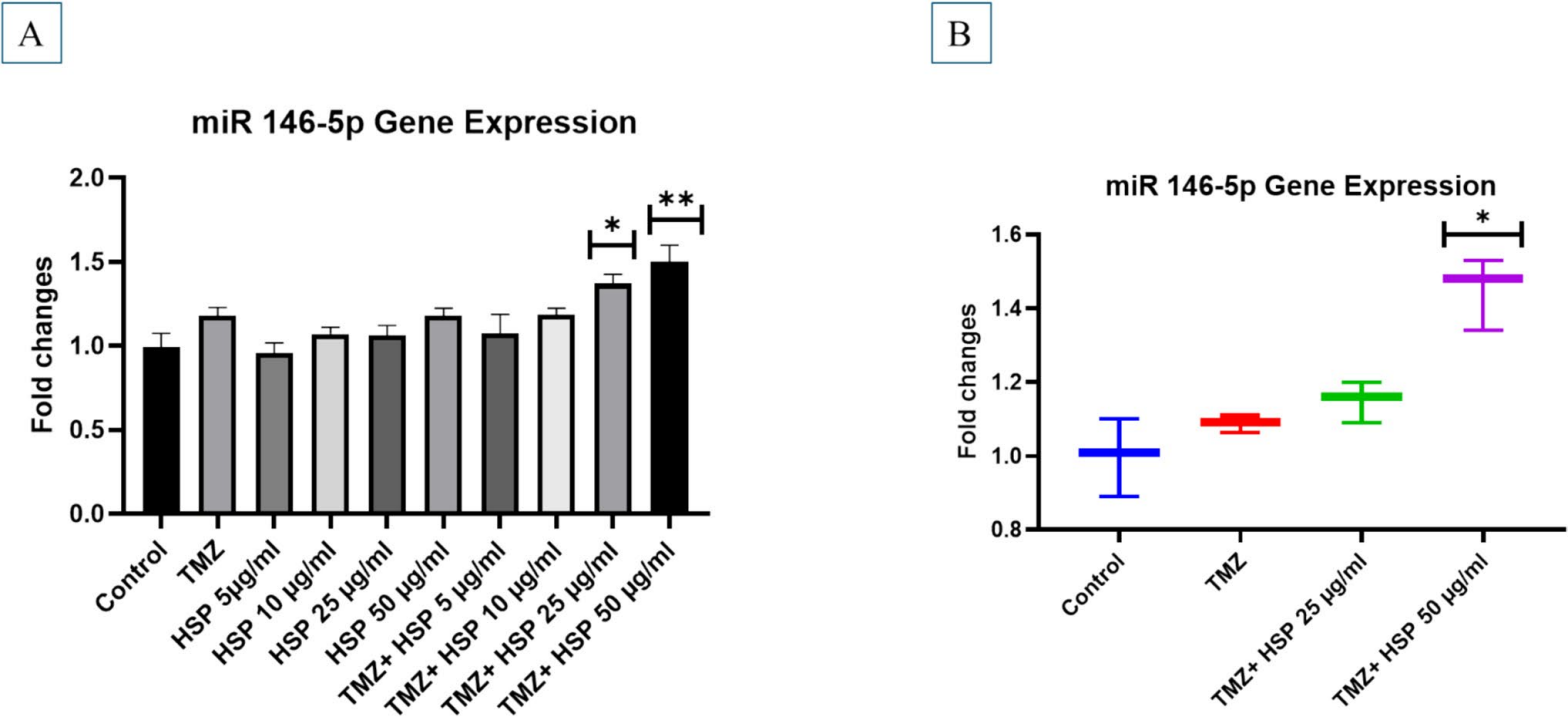
**A** miRNA 146 - 5p gene level of treatment groups. **B** miRNA 146 - 5p gene level of exosomes obtained from treatment groups. Experiments were performed in triplicate, and the results are given as the average. Meaningful statistically: *p < 0.05; **p < 0.01

Our findings show that PDK- 1 and AKT gene levels significantly decreased. Significant downregulation was found in the group treated with 50 µg/mL of TMZ-HSP. A comparable degree of gene-level down-regulation was also seen in exosomes from the investigation groups. PTEN levels, on the other hand, drastically rose in both the treatment groups and their exosomes. This is the first time these data have been compared this way (Fig. 7).

**Fig. 8:**
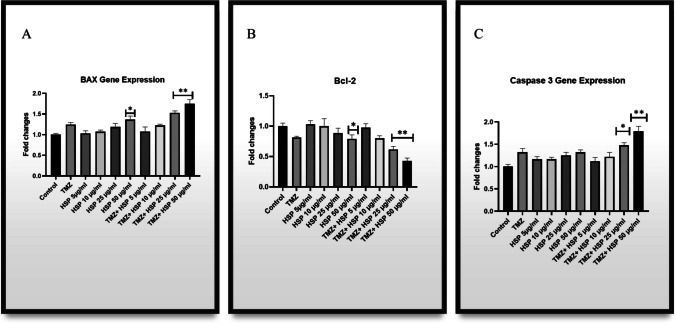
**A** BAX gene level of treatment groups. **B** Bcl- 2 gene level of treatment groups. **C** Caspase 3 gene level of treatment groups. Experiments were performed in triplicate, and the results are given as average. Meaningful statistically: *p < 0.05; **p < 0.01

Furthermore, it has been demonstrated that Bcl- 2 was downregulated; in contrast, Bax levels increased in the experiment groups. The BAX/Bcl- 2 ratio was considerably altered compared to the control group, notably in the groups receiving high doses of TMZ-HSP. Changes in BAX/Bcl- 2 levels led T98G cells to death by activating the Caspase 3 pathway. Our findings are supported by a rise in the expression of the Caspase 3 gene (Fig. 8).

### In human dermal fibroblast cells, HSP healed the wound and enhanced cell viability

**Fig. 9:**
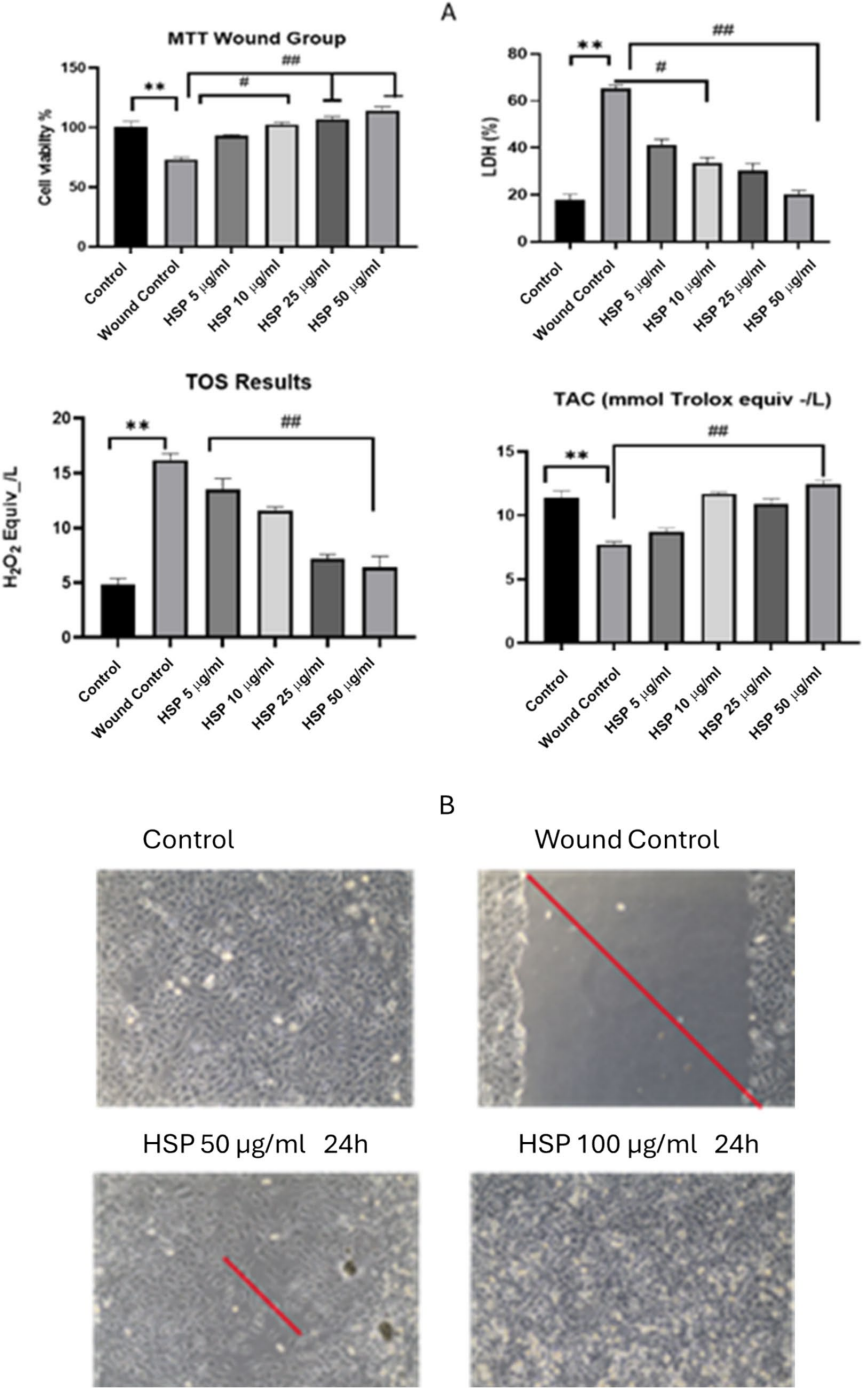
**A** MTT, LDH, TAC, and TOS results of human dermal fibroblast cells. Cell viability decreased, and cytotoxicity increased in the Wound group compared to the control group. Cell viability increased in the groups treated with HSP compared to the wound group. **B** Wound healing figures of the experiment groups. Experiments were performed in triplicate, and the results are given as the average. Meaningful statistically: *p < 0.05; **p < 0.01; *p* < 0.05; ^##^*p* < 0.01

The experiment was terminated after 48 h. When the MTT results of the wound line opened group were examined, it was determined that cell viability decreased by 27%. It was observed that cell viability began to increase in the HSP applied groups. Cell viability was determined to be 114% in the 50 ug/mL group. Considering this information obtained, we determined that HSP has a cell protective effect. Additionally, unlike the wound group, it was found that the LDH level in the HSP groups was similar to the control group. It has been observed that the oxidative damage occurring in the wound line is eliminated by HSP and heals the cells through its antioxidant activity (Fig. 9).

## Discussion

Understanding cancer cell survival mechanisms and the inflammatory tumor microenvironment’s function is crucial to managing the treatment of GB cancer (Sharma et al. 2023). Targeted cancer therapies feature greater susceptibility and an increasingly favorable side effect profile than conventional cytotoxic chemotherapies. In this context, it has been shown that the combination of compounds known to have therapeutic properties with chemotherapeutic drugs increases anticancer efficacy (Gaiaschi et al. 2024). Based on research from numerous in vitro and in vivo settings, HSP has been acknowledged as an effective anti-inflammatory, anti-carcinogenic, and anti-oxidant agent (Devi et al. 2015). To address this objective, we examined how treatment with TMZ-HSP affected T98G cells and T98G cells’ exosomes in this study. The current study reports that TMZ-HSP reduced the PDK- 1/AKT/mTOR gene level and increased the PTEN gene level in the cancer microenvironment. In this respect, our study is important because it is the first study presented in literature. According to our results, TMZ + HES 25 µg/mL and TMZ + HES 50 µg/mL doses can trigger autophagy via the mTOR signaling pathway and apoptosis by activating Caspase 3 via BAX/Bcl- 2. Understanding the cancer cells’ methods of survival and the function of the tumor’s inflammatory microenvironment is crucial to managing the treatment of GB cancer (Røsland & Engelsen 2015; Xiao & Yu 2021; Zhong et al. 2021). HSP’s effectiveness against cancer was evaluated using an examination for cell viability and apoptosis. We also found in this study that HSP protects fibroblast cells and increases cell proliferation. We determined that antioxidant activity increased, especially compared to the wound line group. However, it repaired the wound line completely and increased cell viability beyond the control group. In addition to all of these, it demonstrates that in addition to inducing apoptosis by activating Caspase 3 via BAX/Bcl- 2, it also triggers autophagy via mTOR. Cells started down the apoptotic route when the caspase three gene was activated. The outcomes of our flow cytometry investigations further support our conclusions.

Exosomes transport a variety of signaling molecules and serve as messengers for intercellular communication in addition to their roles in the elimination of extra membranes and the production of damaged proteins and RNAs (Genc et al. 2022; Juan Lu et al. 2018). Therefore, our study demonstrated the effects of exosomes released in T98G cells treated with TMZ-HSP on the autophagic pathway. Tumor-derived exosomes secrete a specific content of miRNA, mRNA, and protein in endothelial cells, thereby altering the process of metastasis and angiogenesis. Exosomes created by tumors generate a unique combination of miRNA, mRNA, and proteins, which regulate angiogenesis and metastasis in endothelial cells (Genc et al. 2023; J Lu et al. 2014). By a variety of examinations, miR- 9 regulates angiogenesis and, with the help of specific target genes, can either promote or prevent endothelial cells (Genc et al. 2022; Madelaine et al. 2017; Huanyu Zhang et al. 2012; Zhuang et al. 2012). A prior investigation demonstrated that miR- 9 might affect the TRIM56/NF-ƘB pathway to encourage the growth of multiple myeloma (Huang et al. 2019). Our study investigating the internal regulatory pathways of TMZ-HSP inhibition of T98G cell activity showed that TMZ-HSP could significantly increase the expression level of miR- 9. In contrast, the current study found that in T98G cells, TMZ-HSP could downregulate the PDK/AKT/mTOR signaling pathway by upregulating miR- 9 expression, thereby affecting the proliferation, migration, invasion, and apoptosis of T98G cells.

mTOR is a protein kinase from the PI3 K-related kinase family. It is heavily involved in cell growth, proliferation, and survival, on the one hand, by enhancing anabolic processes such as protein synthesis and, on the other hand, by suppressing catabolic processes, including autophagy (Hua et al. 2019; Zou et al. 2020). Cellular membrane and protein content disappear due to exosome release and autophagic flux, which are connected processes (Nicklin et al. 2009). According to earlier research, mTOR strictly regulates autophagy in response to nutritional and growth factor circumstances (Nicklin et al. 2009; Tkach & Théry 2016; Trams et al. 1981). Maintaining cellular protein, RNA, and membrane homeostasis requires autophagy and exosome release. While exosome release causes an overall decrease in proteins and membranes, autophagy rescues them to recycle their components. Consequently, maintaining cell fitness under varied circumstances may depend on the harmony between the two processes (Kalluri 2016; Trams et al. 1981). The inclusion of mTORC1 in both processes offers an opportunity for them to coordinate their activities to preserve such a balance.

On the other hand, PTEN (phosphatase and tensin homolog) acts as an essential negative regulator controlling the intracellular levels of PIP3. The PTEN gene is an important tumor suppressor mutated in various cancer types (Di Cristofano & Pandolfi 2000; Jamaspishvili et al. 2018). PTEN catalyzes explicitly the phosphorylation of PIP3, converting it back to PIP2. Recycling of PIP3 to PIP2 acts as a brake on the progression of the PI3 K/AKT/mTOR pathway and plays a vital role in the balance of oncogenic processes in the cell (Manning & Toker 2017). According to our results, there was a significant decrease in PDK- 1 and AKT gene levels. On the contrary, PTEN levels also increased significantly. mTOR is not only an essential effector of cell growth and proliferation, but it can also inhibit autophagy events in its active form. Therefore, inhibiting the expression of P13 K and AKT, which regulate mTOR expression, is a suitable strategy to induce autophagy cell death in GB cells.

Another study that employed IHC to evaluate essential biological aspects of malignant tumors, such as invasion, proliferation, and so on, discovered a link between p53 and GBs mesenchymal subtype. More importantly, the subtypes exhibit distinct differentiation characteristics that point to a relationship to alternate cell types when connected with findings from recent mice research (Jakovlevs et al. 2019). As a result, it provides a foundation for investigating targeted therapeutics. Based on this information, combining HSP and TMZ may have further stimulated the TMZ treatment.

## Conclusions

In conclusion, TMZ-HSP combination decreased Bcl- 2 and increased BAX, released cytochrome-c, and triggered Caspase- 3 in the T98G cell line. In our study, it was determined that HSP had no toxic effect on the fibroblast cell line. On the contrary, it was more effective in wound healing compared to the control group. Similarly, TMZ-HSP combination produced anticancer activity in T98G cells, and this effect also allowed the exosome profiles released from the cells to change. Due to this effect, the TMZ + HSP combination will effectively prevent metastasis in T98G cells. Our study makes an important contribution to literature in all these aspects. On the other hand, further research is needed to determine how exosome contents and/or soluble factors regulate miR- 9, mTOR, and apoptotic pathways in HSP tumor microenvironment.

## Limitations of our study

Our study is limited by the lack of detailed exosome characterization by nanoparticle size distribution (NTA). The findings obtained from our study provide a preliminary study for animal studies. However, the lack of animal studies is among the limitations of our research. However, animal studies are planned to be conducted in future studies and added to the literature.

## Data Availability

All source data for this work (or generated in this study) are available upon reasonable request.
